# Sublethal effects of natural parasitism act through maternal, but not paternal, reproductive success in a wild population

**DOI:** 10.1002/ecy.2772

**Published:** 2019-07-10

**Authors:** Olivia Hicks, Jonathan A. Green, Francis Daunt, Emma J. A. Cunningham, Mark Newell, Adam Butler, Sarah J. Burthe

**Affiliations:** ^1^ School of Environmental Sciences University of Liverpool Nicholson Building Liverpool L69 3BX United Kingdom; ^2^ Centre for Ecology & Hydrology Bush Estate, Penicuik Midlothian EH26 0QB United Kingdom; ^3^ School of Biology Institute of Evolutionary Biology Centre for Immunity, Infection and Evolution University of Edinburgh Ashworth Laboratories King's Buildings, West Mains Road Edinburgh EH9 3JT United Kingdom; ^4^ Biomathematics and Statistics Scotland James Clerk Maxwell Building, The King's Buildings, Peter Guthrie Tait Road Edinburgh EH9 3FD United Kingdom

**Keywords:** breeding success, endoparasites, fitness, life history, macroparasites, maternal, nematodes, parasitism, paternal, reproduction, sex differences, trade‐off

## Abstract

Parasites are a major component of all animal populations. Males and females often differ in their levels of parasite prevalence, potentially leading to sex differences in the impact of parasitism on fitness, with important implications for the evolution of parasite and host traits including resistance, tolerance, and virulence. However, quantitative measures of the impact of parasitism under free‐living conditions are extremely rare, as they require detailed host demographic data with measures of parasite burden over time. Here, we use endoscopy for direct quantification of natural‐parasite burdens and relate these to reproductive success over 7 yr in a wild population of seabirds. Contrary to predictions, only female burdens were associated with negative impacts of parasitism on breeding success, despite males having significantly higher burdens. Female reproductive success declined by 30% across the range of natural parasite burdens. These effects persisted when accounting for interannual population differences in breeding success. Our results provide quantitative estimates of profound sub‐lethal effects of parasitism on the population. Importantly, they highlight how parasites act unpredictably to shape ecological and evolutionary processes in different components of the same population, with implications for demography and selection on host and parasite traits.

## Introduction

Understanding the key drivers of demographic processes is crucial in predicting population growth and persistence (Agnew et al. 2000). Parasites can be one such driver: They are ubiquitous and impact on both survival and reproduction via a suite of lethal and sublethal effects on host behavior, immunity, and resource competition (Lochmiller and Deerenberg 2000). However, individuals may vary in their responses to parasites, and quantifying which components of the population are most affected is key to modeling the effects of parasitism at the population level. There has been particular interest in whether parasitism may operate differently between the sexes: In mammals and birds, for example, males are often more susceptible to parasites and have higher burdens than females, and in invertebrates the opposite relationship tends to be true (Schalk and Forbes 1997, Thompson et al. 2017). This could potentially lead to differential selection by parasitism on males and females, resulting in different levels of resistance and tolerance between sexes (Grossman 1985, Poulin 1996, Schalk and Forbes 1997, Zuk and Stoehr 2002, Duneau and Ebert 2012, Thompson et al. 2017). These effects can lead to different ecological and evolutionary outcomes in different components of a population for a given level of infection (Sheldon and Verhulst 1996, Lochmiller and Deerenberg 2000).

Parasitism can also affect individuals to a different extent because of interactions between extrinsic and intrinsic variables. Levels of individual parasite burdens may interact synergistically with environmental conditions, with impacts of parasitism often more apparent when animals are operating under greater pressure in more marginal conditions (Laaksonen et al. 2002, Tompkins et al. 2011, Granroth‐Wilding et al. 2014). Such interactions may be particularly important during reproduction, because parasites can affect the condition, behavior, and energy use of the host (Sheldon and Verhulst 1996, Binning et al. 2012, Hicks et al. 2018a), thereby limiting the resources available for breeding. It is therefore important to quantify the impact of parasitism on individual reproductive success and how this may interact with varying environmental conditions.

Estimating the impact of parasitism on demographic rates has been challenging, and most studies have employed an experimental approach (Watson 2013). These studies have suggested a significant negative effect of parasitism on host fitness across species with a mean combined effect size *g* of 0.49, though these effects tend to be lower or more difficult to detect in relatively long‐lived species. However, although experimental studies are valuable in untangling effects of parasitism from other covariates such as individual quality, they do not tell us about the relative impact of parasitism between individuals parasitized within the range of natural parasite burdens in the wild. Here we use endoscopy to quantify parasite burdens directly in individuals in a population of European shags (*Phalacrocorax aristotelis*), and relate this to their concurrent breeding success. Endoscopy more accurately reflects absolute parasite burdens than traditional indirect techniques of quantifying parasite load, such as fecal egg counts, which only capture the reproductively active subset of the parasite population (Granroth‐Wilding 2015). Repeat sampling of individuals over time allows the impact of a given parasite burden to be related to breeding success within and between individuals. The study was conducted across a 7‐yr period encompassing a range of environmental conditions, and therefore allowed us to quantify the impact of parasitism on the breeding success of male and female components and age classes of the population.

Specifically, we ask: (1) Does natural parasite load negatively impact individual breeding success? (2) Does parasite load and the effects of parasitism differ between the sexes? (3) Do environmental conditions alter the impact of parasitism on host breeding success?

## Methods

### Measuring parasite load and breeding success

The study was carried out on the Isle of May National Nature Reserve, southeast Scotland (56^°^11′N, 2^°^33′W), during chick rearing in the breeding seasons of 2011–2017. One hundred and one adult European shags were endoscoped to determine natural parasite load of gastrointestinal nematodes *Contracaecum rudoliphii*, a generalist parasite infecting a large number of seabirds. Seabirds can act as the definitive host to *C. rudolphi*, which is transmitted via fish prey (Hoberg 2005). Once consumed by the host, the worms establish in the proventriculus, where they molt to the reproductive adult form (Burthe et al. 2013). Fifty‐two adult host shags were endoscoped in at least 2 yr, giving a total sample size of 220 observations (106 male, 114 female; see Appendix S1: Table S1 for details of sampling structure). Consistent with previous work, endoscopy revealed all adults were parasitized, but that burdens varied both between individuals and within individuals across years, which implies that there is potential to separate out the effects of parasite burdens from variations in individual quality. Worm burdens were counted visually, using video images from the endoscope (for details see Burthe et al. 2013, Hicks et al. 2018a, b). This method provides a more representative measure of burden than indirect methods, such as fecal egg counts, which significantly underestimate levels of parasitism, as they only detect sexually mature adult worms that are producing eggs (Granroth‐Wilding et al. 2016). One measure of parasite load was taken for individuals within each year, which has been shown to be repeatable across the breeding season (Burthe et al. 2013). All individual shags were uniquely marked with a metal ring as chicks (therefore of known age) and sexed by vocalization (Snow 1960). In shags both the male and female take turns incubating the eggs and provisioning the young, and breeding success, number of chicks fledged per pair, was recorded for all individuals. All endoscopy was performed by trained personnel (S. Burthe) holding a personal license operating under a project license issued by the U.K. Home Office under the Animals (Scientific Procedures) Act 1986. We used mean population productivity as an annual proxy for environmental conditions (as in Reed et al. 2008b, Granroth‐Wilding et al. 2014, Bogdanova et al. 2014). This was calculated as the average number of fledged young per incubated nest in a series of unmanipulated, long‐term monitoring plots completely independent of the birds included in the parasite study (see Newell et al. 2015 for monitoring methods). This long‐term monitoring program also allowed us to estimate mean population lay date. The span of data collection beyond the dates of the present study also allowed us to include lag mean population productivity in some analyses.

### Statistical analysis

All models were linear mixed models or generalized linear mixed models (GLMMs), and were fitted using the *lme4* package in R (Bates et al. 2014, R Core Team 2015). We controlled for variation between birds and for repeated sampling by including individual as a random effect in all models. *P* values were calculated using likelihood‐ratio tests, as implemented in R using the ‘anova,’ function and are provided for all analyses.

To test for a difference in parasite loads between sexes (Q2) we compare linear mixed models describing parasite load (worm count) with and without the term sex using a likelihood‐ratio test, while accounting for the explanatory variables age, a quadratic effect of age and year (as a categorical variable). After carrying out a preliminary analysis on the relationship between parasite load and body mass, it was not included as an explanatory variable in these models; see Appendix S2 for details. These models assumed that parasite load was normally distributed, which was the best approximation for the complex empirical distribution of these data.

In all other models, the response variable was the number of chicks fledged, relative to the maximum number of eggs laid, representing “fledging probability per egg,” which was assumed to have a binomial distribution (with logit link function). For these analyses, we fitted models for males and females separately, because of nonindependence of nest pairs, to quantify the impact of parasitism on the breeding success of both sexes directly. Preliminary analysis showed no correlation between male and female parasite load from the same nest in the same year (*r* = 0.083, *n* = 34).

To answer Q1, that is, whether natural parasite burdens negatively impact the breeding success of their hosts, we used a likelihood‐ratio test to compare a model containing explanatory variables that may affect breeding success: age, quadratic effect of age (Daunt et al. 1999, Jaeger et al. 2014), and year (as a categorical variable), which accounted for known interannual variability, against the same model with the addition of individual parasite load. To answer Q3, that is, whether parasite load interacts with extrinsic variables to impact breeding success, we considered a null model that contains age, quadratic effect of age, and variables that represent the extrinsic environment: mean population productivity, mean lay date, and lag mean population productivity. We then used likelihood‐ratio tests to calculate the *P* values associated with adding interactions between each of these variables and parasite load to the null model.

## Results

**Figure 1:**
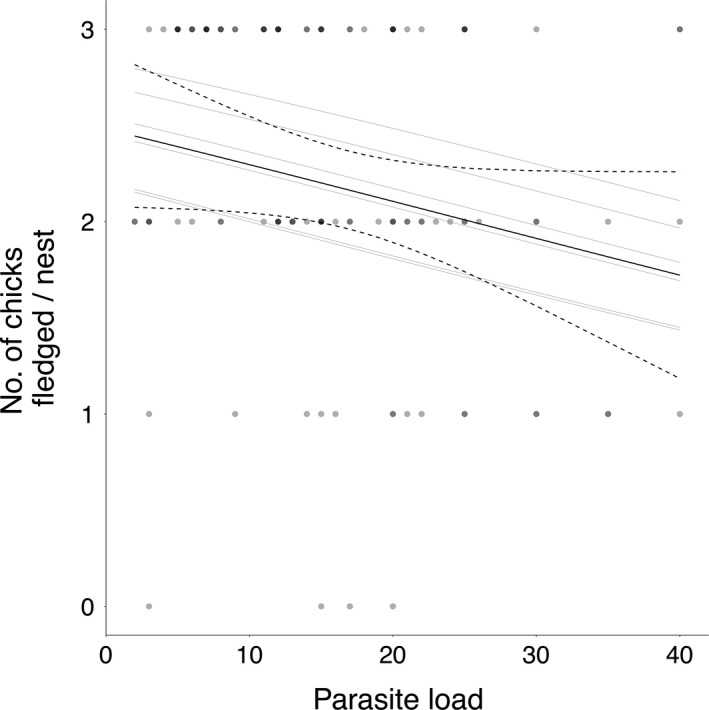
The effect of parasite load on breeding success (number of chicks raised per nest) in female European shags. Lines represent predicted lines from the best supported model (solid lines). Gray lines represent the predicted lines for each year of the study, which vary in the mean population productivity (a proxy for environmental conditions). The black line represents the predicted line under the mean environmental conditions with 95% confidence intervals (dashed). Points are shaded based on density for ease of interpretation of the underlying data.

Parasite loads included in these analyses ranged from 2 to 40 worms, though these distributions differed between the sexes—males mean: 23, SD ± 11.0, females mean: 16, SD ± 9.2—likelihood‐ratio test: *χ* ^2^ = 17.93; *P* = <0.001. Despite males being more heavily parasitized, we found that individual fledgling success was negatively related to parasite load in females but not males. For females, the addition of parasite load to the model significantly improved the fit of the model (*χ* ^2^ = 4.56; *P* = 0.03) and led to predicted breeding success values that were 30% lower at the highest observed parasite loads than at the lowest observed loads, equivalent to a loss of 0.7 chicks per adult (see Fig. 1). For males, the addition of parasite load did not improve the model (*χ* ^2^ = 0.15; *P* = 0.70) and although there was evidence for a significant quadratic effect of age on the number of chicks fledged (*χ* ^2^ = 7.46; *P* = 0.01; see Appendix S1: Table S3) there was no interaction between age and parasite burden on breeding success (see Appendix S1: Fig. S2).

There was no interaction between parasitism and the prevailing environment in driving breeding success for either females or males, as including interactions between parasitism and any of the environmental variables did not improve the model fit (see Tables 1 and 2). This suggests that for females, the negative effect of parasitism on fitness is persistent across all conditions experienced by birds in the study.

**Table 1:** The relationship between breeding success, parasite load, and extrinsic variables in female European shags

Explanatory variable	*P* value	Effect size	±SE	Question
Parasite load	**0.04**	−0.22	0.11	Q1
Parasite load	**0.03**	−0.23	0.10	Q3
Parasite load × mean productivity	0.30	0.12	0.12	
Parasite load × lag prod	0.93	0.03	0.10	
Parasite load × lay date	0.28	−0.16	0.12	
Parasite load × age	0.45	−0.08	0.11	
Parasite load × age^2^	0.67	0.05	0.09	

*Notes*

Models predicting individual‐level breeding success (number of chicks fledged per nest), with response variables parasite load, mean population productivity, mean lag population productivity, mean lay date, and adult age, and the interactions between these variables and parasite load. Estimates from generalized linear mixed models of effect sizes and estimates are presented for all effects. *P* values were calculated using likelihood‐ratio tests. Statistically significant terms are indicated in bold.

**Table 2:** The relationship between breeding success, parasite load, and extrinsic variables in male European shags

Explanatory variable	*P* value	Effect size	±SE	Question
Parasite load	0.70	−0.04	0.11	Q1
Parasite load	0.44	−0.08	0.11	Q3
Parasite load × mean productivity	0.22	−0.13	0.11	
Parasite load × lag prod	0.86	0.02	0.10	
Parasite load × lay date	0.79	0.03	0.10	
Parasite load × age	0.56	−0.07	0.11	
Parasite load × age^2^	0.82	−0.04	0.16	

*Notes*

Models predicting individual‐level breeding success (number of chicks fledged per nest), with response variables parasite load, mean population productivity, mean lag population productivity, mean lay date, and adult age, and the interactions between these variables and parasite load. Estimates from general linear mixed models of effect sizes and estimates are presented for all effects. *P* values presented were calculated from likelihood‐ratio tests between models with and without the term of interest.

## Discussion

We found parasite burden had a significant impact on the reproductive success of female free‐living shags. However, despite having significantly higher burdens, the same relationship was not found in males. We found no evidence of the environment mediating the effects of parasitism in either sex. This suggests that the negative fitness effects of parasitism are additive rather than interactive with environmental conditions and operate even under favorable conditions.

It is unusual to be able to show such clear fitness impacts of natural parasite burdens (though see Hayward et al. 2014), with most studies having taken an experimental approach (e.g., see Watson 2013, Hudson et al. 1998, Albon et al. 2002, including this study system, Reed et al. 2008a). However, we found a 30% decline in host fledgling success across the natural range of parasite load, which is important, as it shows that natural ranges of parasitism existing in wild populations are exerting significant sublethal fitness effects. This effect is particularly notable given that relatively long‐lived species, such as shags, tend to display lesser effects of parasitism than short‐lived hosts (Watson 2013). The costs were only detected in females, contrary to the prediction that males, due to their higher parasite burdens, pay a greater cost of parasitism than females (Grossman 1985, Poulin 1996, Zuk and Stoehr 2002, Duneau and Ebert 2012, Thompson et al. 2017). However, the findings are consistent with the idea that selection may act differently on the sexes to influence traits that affect the ability to cope with a given level of parasitism. Different levels of exposure or sex‐specific physiology may lead to differences in the strength of selection or the traits upon which selection may act, resulting in differing levels of resistance or tolerance for a given parasite load between the sexes (Duneau and Ebert 2012, Thompson et al. 2017).

Although our analysis is necessarily correlative in order to measure the impact of parasitism within the range of natural variation, our results combined with previous experimental studies support the effect of parasitism as being a causal factor of poor reproductive performance: First, repeat sampling of the same individuals across years allows us to test the impact on the same individual with different parasite loads and second, experimental removal of parasitism improves both foraging and breeding performance (Reed et al. 2008a). Our results are also consistent with previous work in this species, showing the energetic and behavioral costs of natural parasitism in females but not males (Hicks et al. 2018a *,* b).

Considering these results from an energetic perspective can help to elucidate potential mechanisms by which parasites negatively affect the host. Highly parasitized female shags are known to experience higher resting metabolic rates and higher flight costs, meaning foraging and crucially provisioning costs are more energetically demanding (Hicks et al. 2018a, b). This could lead to the reduced breeding success we see in more highly parasitized individuals, given the increased cost to raising young efficiently. Female shags also experience a larger effect of wind on their year‐round foraging behavior than males, and highly parasitized females experience increased flight costs, suggesting they are more susceptible to extrinsic drivers than males, conceivably the reason there are measurable effects of parasitism to females and not males (Lewis et al. 2015, Hicks et al. 2018a).

We also found sex‐specific age effects; males showed a quadratic relationship between age and breeding success (with the highest breeding success occurring at intermediate ages, and lower breeding success in the youngest and oldest birds), but females did not. This occurs in a number of wild vertebrate populations and is well documented in seabirds (Daunt et al. 1999, Froy et al. 2017, Clay et al. 2018). The effect of age on breeding success found in males could be in part linked to their response to parasitism. Males may bear the cost of parasitism rather than invest in an immune response, but this may cause long‐term somatic damage (Hasselquist and Nilsson 2012), contributing to their decline in breeding success in later life. Crucially these differing effects of parasitism on males and females may have significant implications for selection and population processes.

Effects of parasitism are often context and host‐condition dependent, with effects expected to be more apparent in bad conditions (Bustnes et al. 2006, Bize et al. 2010, Granroth‐Wilding et al. 2014). Previous experimental work with this system found that for chicks, the effects of parasite removal were more pronounced in years of poor productivity (Granroth‐Wilding et al. 2014). Yet we found no evidence for an interaction between parasitism and environmental conditions in their effects on reproduction in adults. This suggests that parasitism will not just impact individuals under poor conditions as predicted, but substantially reduce reproductive output across all years, and hence have a greater impact on population dynamics than previously thought. However, it is important to note that over the study, conditions were always relatively good when compared to long‐term data from this population, suggesting our estimates of impact may be conservative.

In conclusion, our results demonstrate that although parasite burden may be associated with differences in breeding success within the population, the direction or strength of this relationship can differ between sexes. They also highlight that although one sex may be more heavily parasitized than another, this does not translate directly into parasitism impacts. Differences between the sexes may have led to different selective pressures affecting the ability of individuals to tolerate a given level of infection (Duneau and Ebert 2012, Thompson et al. 2017) leading to differences in how demographic traits are affected. Integrating these differences into demographic models will be essential in calculating the likely impact of parasitism of populations.

## References

[ecy2772-bib-0001] Agnew, P. , J. C. Koella , and Y. Michalakis . 2000 Host life history responses to parasitism. Microbes and Infection 2:891–896. 10962272 10.1016/s1286-4579(00)00389-0

[ecy2772-bib-0002] Albon, S. D. , A. Stien , R. J. Irvine , R. Langvatn , E. Ropstad , and O. Halvorsen . 2002 The role of parasites in the dynamics of a reindeer population. Proceedings of the Royal Society B 269:1625–1632. 12184833 10.1098/rspb.2002.2064 PMC1691070

[ecy2772-bib-0003] Bates, D. , M. Mächler , B. Bolker , and S. Walker . 2014 Fitting linear mixed‐effects models using lme4. Journal of Statistical Software 67:1–48.

[ecy2772-bib-0004] Binning, S. A. , D. G. Roche , and C. Layton . 2012 Ectoparasites increase swimming costs in a coral reef fish. Biology Letters 9:20120927. 10.1098/rsbl.2012.0927 PMC3565510 23193046

[ecy2772-bib-0005] Bize, P. , R. Piault , J. Gasparini , and A. Roulin . 2010 Indirect costs of parasitism are shaped by variation in the type of immune challenge and food availability. Evolutionary Biology 37:169–176.

[ecy2772-bib-0006] Bogdanova, M. I. , S. Wanless , M. P. Harris , J. Lindström , A. Butler , M. A. Newell , K. Sato , Y. Watanuki , M. Parsons , and F. Daunt . 2014 Among‐year and within‐population variation in foraging distribution of European shags *Phalacrocorax aristotelis* over two decades: Implications for marine spatial planning. Biological Conservation 170:292–299.

[ecy2772-bib-0007] Burthe, S. J. , M. A. Newell , G. Goodman , A. Butler , T. Bregnballe , E. Harris , S. Wanless , E. J. A. Cunningham , and F. Daunt . 2013 Endoscopy as a novel method for assessing endoparasite burdens in free‐ranging European shags (*Phalacrocorax aristotelis*). Methods in Ecology and Evolution 4:207–216.

[ecy2772-bib-0008] Bustnes, J. O. , K. E. Erikstad , S. A. Hanssen , T. Tveraa , I. Folstad , and J. U. Skaare . 2006 Anti‐parasite treatment removes negative effects of environmental pollutants on reproduction in an Arctic seabird. Proceedings of the Royal Society B 273:3117–3122. 17015342 10.1098/rspb.2006.3687 PMC1679894

[ecy2772-bib-0009] Clay, T. A. , E. J. Pearmain , R. A. R. McGill , A. Manica , and R. A. Phillips . 2018 Age‐related variation in non‐breeding foraging behaviour and carry‐over effects on fitness in an extremely long‐lived bird. Functional Ecology 50:700.

[ecy2772-bib-0010] Daunt, F. , S. Wanless , M. P. Harris , and P. Monaghan . 1999 Experimental evidence that age‐specific reproductive success is independent of environmental effects. Proceedings of the Royal Society B 266:1489–1493.

[ecy2772-bib-0011] Duneau, D. , and D. Ebert . 2012 Host sexual dimorphism and parasite adaptation. PLoS Biology 10:1–9. 10.1371/journal.pbio.1001271 PMC3289593 22389630

[ecy2772-bib-0012] Froy, H. , S. Lewis , D. H. Nussey , A. G. Wood , and R. A. Phillips . 2017 Contrasting drivers of reproductive ageing in albatrosses. Journal of Animal Ecology 86:1022–1032. 28605018 10.1111/1365-2656.12712 PMC5601251

[ecy2772-bib-0013] Granroth‐Wilding, H. M. V. , S. J. Burthe , S. Lewis , T. E. Reed , K. A. Herborn , M. A. Newell , E. A. Takahashi , F. Daunt , and E. J. A. Cunningham . 2014 Parasitism in early life: Environmental conditions shape within‐brood variation in responses to infection. Ecology and Evolution 4:3408–3419. 25535557 10.1002/ece3.1192 PMC4228615

[ecy2772-bib-0038] Granroth‐Wilding, H. M. V. , S. J. Burthe , S. Lewis , K. A. Herborn , E. A. Takahashi , F. Daunt , and E. J. A. Cunningham . 2015 Indirect effects of parasitism: costs of infection to other individuals can be greater than direct costs borne by the host. Proceedings Biological Sciences 282: pii 20150602. 10.1098/rspb.2015.0602 PMC4528545 26156765

[ecy2772-bib-0014] Granroth‐Wilding, H. M. V. , F. Daunt , E. J. A. Cunningham , and S. J. Burthe . 2016 Between‐individual variation in nematode burden among juveniles in a wild host. Parasitology:1–11. 10.1017/S0031182016001700 27873556

[ecy2772-bib-0015] Grossman, C. J. 1985 Interactions between the gonadal steroids and the immune system. Science 227:257–261. 3871252 10.1126/science.3871252

[ecy2772-bib-0016] Hasselquist, D. , and J. Å. Nilsson . 2012 Physiological mechanisms mediating costs of immune responses: What can we learn from studies of birds? Animal Behaviour 83:1303–1312.

[ecy2772-bib-0017] Hayward, A. D. , D. H. Nussey , A. J. Wilson , C. Berenos , J. G. Pilkington , K. A. Watt , J. M. Pemberton , and A. L. Graham . 2014 Natural selection on individual variation in tolerance of gastrointestinal nematode infection. PLoS Biology 12:1–13. 10.1371/journal.pbio.1001917 PMC4114752 25072883

[ecy2772-bib-0018] Hicks, O. , S. J. Burthe , F. Daunt , M. Newell , A. Butler , M. Ito , K. Sato , and J. A. Green . 2018a The energetic cost of parasitism in a wild population. Proceedings of the Royal Society B 285:20180489. 29848646 10.1098/rspb.2018.0489 PMC5998108

[ecy2772-bib-0019] Hicks, O. , S. J. Burthe , F. Daunt , M. Newell , O. Chastel , C. Parenteau , and J. A. Green . 2018b The role of parasitism in the energy management of a free‐ranging bird. Journal of Experimental Biology 221:jeb190066. 30397174 10.1242/jeb.190066 PMC6307876

[ecy2772-bib-0020] Hoberg, E. P. 2005 In K. Rohde, editor, Marine parasitology. Marine birds and their helminth parasites (114–120) Csiro Publishing, Clayton, Australia.

[ecy2772-bib-0021] Hudson, P. J. , A. Dobson , and D. Newborn . 1998 Prevention of population cycles by parasites removal. Science 282:2256–2258. 9856948 10.1126/science.282.5397.2256

[ecy2772-bib-0022] Jaeger, A. , A. Goutte , V. J. Lecomte , P. Richard , O. Chastel , C. Barbraud , H. Weimerskirch , and Y. Cherel . 2014 Age, sex, and breeding status shape a complex foraging pattern in an extremely long‐lived seabird. Ecology 95:2324–2333. 25230482 10.1890/13-1376.1

[ecy2772-bib-0023] Laaksonen, T. , E. Korpimaki , and H. Hakkarainen . 2002 Interactive effects of parental age and environmental variation on the breeding performance of Tengmalm's owls. Journal of Animal Ecology 1:23–31.

[ecy2772-bib-0024] Lewis, S. , R. A. Phillips , S. J. Burthe , S. Wanless , and F. Daunt . 2015 Contrasting responses of male and female foraging effort to year‐round wind conditions. Journal of Animal Ecology 84:1490–1496. 26283625 10.1111/1365-2656.12419 PMC4989534

[ecy2772-bib-0025] Lochmiller, R. L. , and C. Deerenberg . 2000 Trade‐offs in evolutionary immunology: just what is the cost of immunity? Oikos 88:87–98.

[ecy2772-bib-0026] Newell, M. , S. Wanless , M. Harris , and F. Daunt . 2015 Effects of an extreme weather event on seabird breeding success at a North Sea colony. Marine Ecology Progress Series 532:257–268.

[ecy2772-bib-0027] Poulin, R. 1996 Sexual inequalities in helminth infections: a cost of being a male? American Naturalist 2:420–422.

[ecy2772-bib-0028] R Development Core Team . 2015 R: A language and environment for statistical computing. R Foundation for Statistical Computing, Vienna, Austria http://www.r-project.org

[ecy2772-bib-0029] Reed, T. E. , F. Daunt , M. E. Hall , R. A. Phillips , S. Wanless , and E. J. A. Cunningham . 2008a Parasite treatment affects maternal investment in sons. Science 321:1681–1682. 18687923 10.1126/science.1159466

[ecy2772-bib-0030] Reed, T. E. , L. E. B. Kruuk , S. Wanless , M. Frederiksen , E. J. A. Cunningham , and M. P. Harris . 2008b Reproductive senescence in a long‐lived seabird: rates of decline in late‐life performance are associated with varying costs of early reproduction. American Naturalist 171:E89–E101. 10.1086/524957 18173366

[ecy2772-bib-0031] Schalk, G. , and M. R. Forbes . 1997 Male biases in parasitism of mammals: effects of study type, host age, and parasite taxon. Oikos 78:67–74.

[ecy2772-bib-0032] Sheldon, B. C. , and S. Verhulst . 1996 Ecological immunology: costly parasite defences and trade‐offs in evolutionary ecology. Trends in Ecology and Evolution 11:317–321. 21237861 10.1016/0169-5347(96)10039-2

[ecy2772-bib-0033] Snow, B. 1960 The breeding biology of the shag *Phalacrocorax aristotelis* on the Island of Lundy, Bristol Channel. Ibis 102:554–575.

[ecy2772-bib-0034] Thompson, O. , S. A. Y. Gipson , and M. D. Hall . 2017 The impact of host sex on the outcome of co‐infection. Scientific Reports 7:1–7. 28424526 10.1038/s41598-017-00835-z PMC5430432

[ecy2772-bib-0035] Tompkins, D. M. , A. M. Dunn , M. J. Smith , and S. Telfer . 2011 Wildlife diseases: From individuals to ecosystems. Journal of Animal Ecology 80:19–38. 20735792 10.1111/j.1365-2656.2010.01742.x

[ecy2772-bib-0036] Watson, M. J. 2013 What drives population‐level effects of parasites? Meta‐analysis meets life‐history. International Journal for Parasitology: Parasites and Wildlife 2:190–196 . 24533334 10.1016/j.ijppaw.2013.05.001 PMC3862538

[ecy2772-bib-0037] Zuk, M. , and A. M. Stoehr . 2002 Immune defense and host life history. American Naturalist 160:S9–S22. 10.1086/342131 18707455

