# Supplementary material for: Sublethal effects of natural parasitism act through maternal, but not paternal, reproductive success in a wild population
Source: Ecology. 2019 Jul 10;100(8):e02772. doi: 10.1002/ecy.2772 (PMC6851849; doi:10.1002/ecy.2772)
Supplement: Supplementary file 1 [file ECY-100-na-s001.pdf]

Supporting Information: Olivia Hicks, Jonathan A. Green, Francis Daunt, Emma Cunningham, Mark Newell, Adam Butler, Sarah J. Burthe. 2019. Sub-lethal effects of natural parasitism act through maternal but not paternal reproductive success in a wild population. *Ecology.*

Appendix S1

Table S1. The number of unique individual adult European shags sampled in multiple years over the duration of the seven-year study. Sampling included measuring endo-parasite load using an endoscope technique and recording reproductive success (fledglings raised) for each individual.

**No. years sampled**	**6**	**5**	**4**	**3**	**2**	**1**
**Count**	4	4	15	9	20	49
**Cumulative count**	4	8	23	32	52	101

Table S2. The relationship between breeding success, parasite load and extrinsic variables in male and female European shags. Models predicting individual level breeding success (number of chicks fledged per nest), with response variables parasite load, mean population productivity, mean lag population productivity, mean lay date and adult age and the quadratic effect of age. Estimates from general linear mixed models of slopes and estimates are presented for all effects. P values presented from likelihood ratio tests between models with and without the term of interest. Statistically significant terms are indicated in bold

	**Explanatory variable**	**P Value**	**Effect size**	**±SE**
Females	Age	0.99	0.00	0.13
	Age^2	0.08	0.12	0.07
	**Mean productivity**	**<0.01**	0.52	0.17
	Lag productivity	0.31	-0.13	0.13
	Lay date	0.15	0.29	0.20
	**Parasite load**	**0.03**	-0.23	0.10
Males	Age	0.09	0.18	0.11
	Age^2	0.67	0.04	0.08
	Mean productivity	0.12	0.25	0.16
	Lag productivity	0.48	-0.09	0.13
	Lay date	0.49	0.13	0.18
	Parasite load	0.44	-0.08	0.11

Table S3. The relationship between breeding success, parasite load and extrinsic variables in male and female European shags. Models predicting individual level breeding success (number of chicks fledged per nest), with response variables parasite load, breeding year, adult age and the quadratic effect of age. Estimates from general linear mixed models of slopes and estimates are presented for all effects. These outputs are from analysis used to answer Q1, but provide output for all variables included in the model in addition to parasite load. P values presented from likelihood ratio tests between models with and without the term of interest. Statistically significant terms are indicated in bold

	Explanatory variable	P-Value	Effect size	±SE
Female	Age	0.35	0.14	0.15
	Age^2	0.92	-0.01	0.10
	Year 2011	0.22	-0.32	0.26
	Year 2012	0.94	-0.05	0.62
	Year 2013	0.64	0.15	0.31
	**Year 2014**	**0.04**	0.57	0.27
	Year 2015	0.05	0.47	0.24
	**Year 2016**	**0.01**	0.66	0.26
	Year 2017	0.48	0.19	0.26
	**Parasite load**	**0.04**	-0.22	0.11
Male	**Age**	**0.02**	0.26	0.11
	**Age^2**	**0.01**	-0.33	0.13
	Year 2011	0.38	0.25	0.29
	Year 2012	0.31	0.38	0.38
	Year 2013	0.10	0.45	0.27
	Year 2014	0.01	0.93	0.36
	**Year 2015**	**0.01**	0.68	0.27
	**Year 2016**	**<0.01**	0.98	0.31
	**Year 2017**	**0.03**	0.59	0.27
	Parasite load	0.70	-0.04	0.11

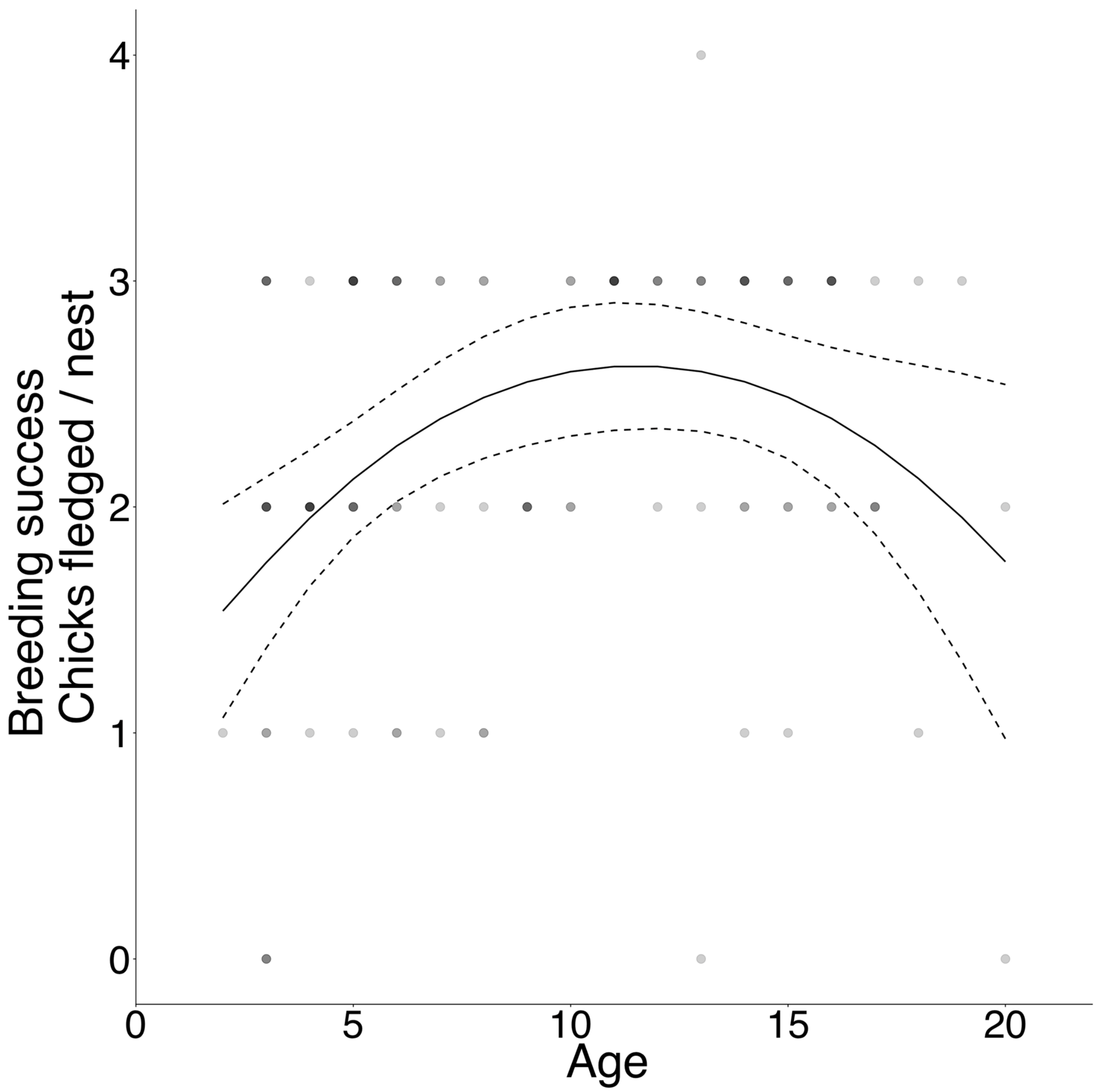

Fig S1. The effect of adult age on breeding success (number of chicks raised per nest) of male European shags. Lines show predicted lines (solid line) and their 95% confidence intervals (dashed) from the best supported model to describe individual variation in breeding success. Points are shaded based on density for ease of interpretation of the underlying data.

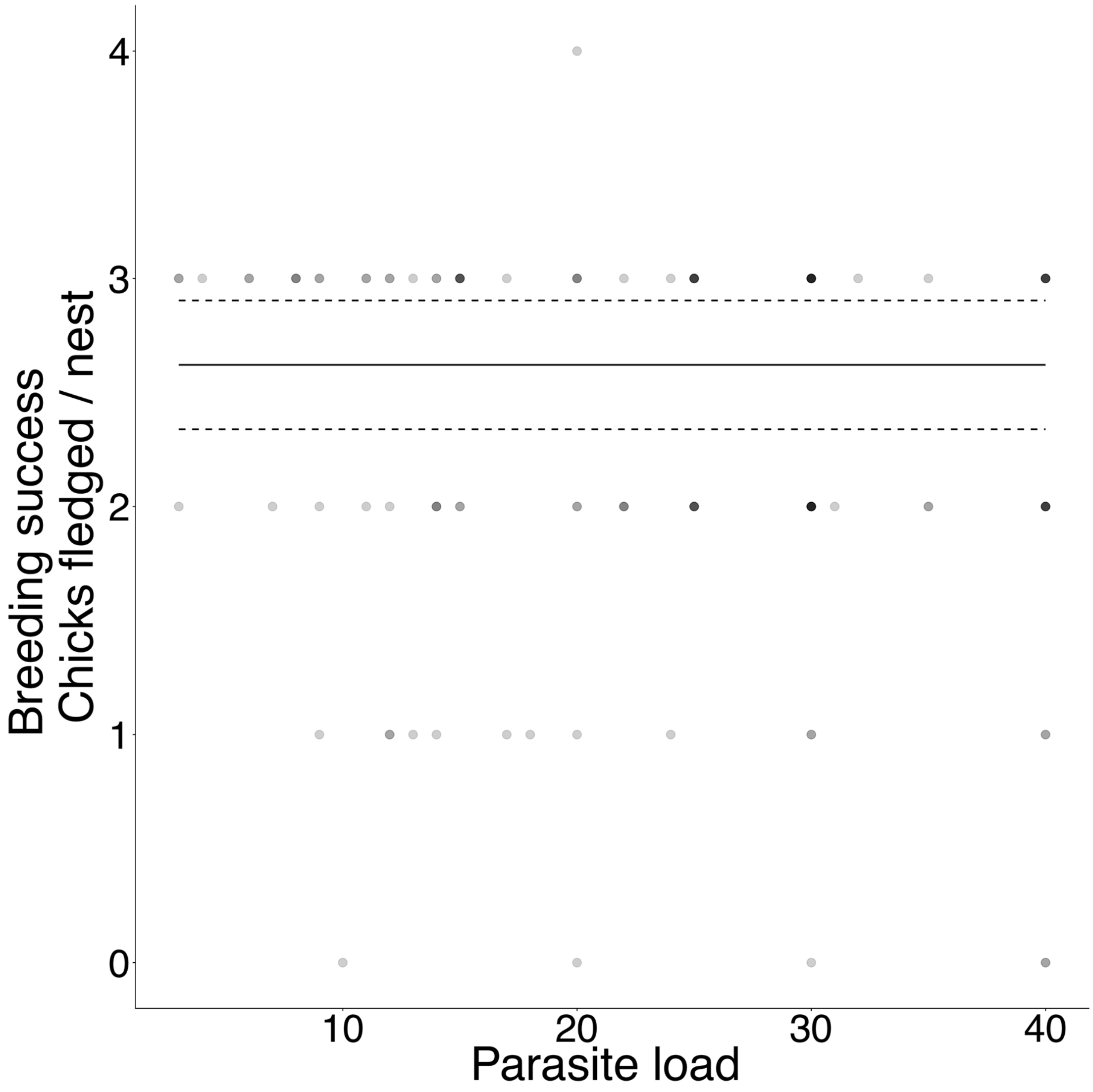

Fig S2. The effect of parasite load on breeding success (number of chicks raised per nest) in male European shags. Lines represent predicted lines from the best supported model (solid lines). Grey lines represent the predicted lines for each year of the study which vary in the mean population productivity (a proxy for environmental conditions), the black line represents the predicted line under the mean environmental conditions with 95% confidence intervals (dashed). Points are shaded based on density for ease of interpretation of the underlying data.
