# Supplementary material for: Sublethal effects of natural parasitism act through maternal, but not paternal, reproductive success in a wild population
Source: Ecology. 2019 Jul 10;100(8):e02772. doi: 10.1002/ecy.2772 (PMC6851849; doi:10.1002/ecy.2772)
Supplement: Supplementary file 2 [file ECY-100-na-s002.pdf]

Supporting Information: Olivia Hicks, Jonathan A. Green, Francis Daunt, Emma Cunningham, Mark Newell, Adam Butler, Sarah J. Burthe. 2019. Sub-lethal effects of natural parasitism act through maternal but not paternal reproductive success in a wild population. *Ecology.*

Appendix S2

The effect of body mass on parasite load and breeding success

Shags and cormorants store very little fat on their bodies, meaning that it is not very easy to measure body condition in these species. However since body mass is often a good measure in quality and condition in seabird species we investigated the use of this as a proxy for quality in this host system. As body mass data only exists for a smaller portion of the total parasite dataset these analyses helped to understand whether this variable should be used in the main analysis of the manuscript. Using a subset of the dataset from 2014-2017 for which individual body mass data exist, we investigated the influence of body mass on parasite load and breeding success. We used a likelihood ratio test to compare models describing parasite load based on explanatory variables that may affect parasite load: age, quadratic effect of age and mean population productivity, against the same model with the addition of individual mass.

The addition of mass to a model describing parasite load did not significantly improve the model (females: $\chi^2 = 0.54$; p= 0.9, males: $\chi^2 = 1.05$; p= 0.31).

To investigate the possible influence of body mass on breeding success we used models where the response variable was the number of chicks fledged, relative to the maximum number of eggs laid, representing 'fledging probability per egg', which was assumed to have a binomial distribution (with logit link function). We used a likelihood ratio test to compare models with explanatory effects that may affect breeding success: parasite load, age, quadratic effect of age and year (as a categorical variable), which accounted for known inter-annual variability, against the same model with the addition of individual mass. The addition of mass to the models describing breeding success for male and females did not significantly improve the models (females: $\chi^2 = 2.10$; p= 0.15, males: $\chi^2 = 3.28$; p= 0.07).
